# Homocysteine and C-Reactive Protein Associated with Progression and Prognosis of Intracranial Branch Atheromatous Disease

**DOI:** 10.1371/journal.pone.0073030

**Published:** 2013-09-11

**Authors:** Xuejiao Men, Jiejie Li, Bingjun Zhang, Lei Zhang, Haiyan Li, Zhengqi Lu

**Affiliations:** Department of Neurology, The Third Affiliated Hospital of Sun Yat-Sen University, Guangzhou, Guangdong, People's Republic of China; University of Campinas, Brazil

## Abstract

**Background and Objectives:**

C-reactive protein (CRP) is a biomarker of inflammation and a sensitive predictor of stroke, and high homocysteine (Hcy) is also associated with stroke. However, the roles of CRP and Hcy in the pathogenesis, progression and prognosis of branch atheromatous disease (BAD) and lipohyalinotic degeneration (LD) are largely unknown. We sought to determine the relation between them in Chinese patients.

**Methods:**

According to the lesion presences shown by diffusion-weighted imaging (DWI), we retrospectively recruited a cohort of 308 patients with a diagnosis of BAD and LD from a total of 1458 consecutive patients with acute ischemic stroke. Progression was defined as worsening by > or  = 1-point in the NIHSS for motor function within the first 5 days. Good outcome was deemed as Modified Rankin Scale (mRS) ≤ 2 and poor outcome was mRS > 2 recorded at one month after onset.

**Results:**

This study comprised a total of 179 patients with BAD and 129 patients with LD. Subjects in patients with LD significantly had an elevated Hcy (p = 0.030), a lower NIHSS score on admission (p<0.001) and mRS score at one month after ictus (p<0.001) than those in patients with BAD. Elevated Hcy (P = 0.004) and increased CRP (P = 0.025) were associated with progression in patients with BAD, and CRP (p = 0.006) and diabetes mellitus (p = 0.011) were found to be associated with poor outcome in patients with BAD. However, no association was observed in patients with LD on progression and prognosis. After multivariate logistic regression analysis, elevated Hcy (p = 0.002) remained the only independent predictor for the progression, and increased CRP (p = 0.027) and smoking (p = 0.012) became the independent predictors for the poor outcome in patients with BAD.

**Conclusions:**

In patients with BAD, elevated Hcy and increased CRP may independently predict progression and prognosis, respectively.

## Introduction

In recent years, intracranial branch atheromatous disease (BAD), a common etiological category of acute ischemic stroke (AIS) in Asian populations, characterized by progressive motor deficits in early acute phase, has drawn much more attention than before. The pathogenesis of BAD probably starts with atherogenesis at the vessel orifices of lager caliber penetrating arteries and is different from lipohyalinotic degeneration (LD), which is caused by lipohyalinotic degenerative changes of the terminal penetrating arteries [1]–[3]. However, there are accumulating evidences suggesting that atherogenesis is inflammatory condition [4]. Inflammation, an important factor in ischemic stroke, to a certain extent, is not only playing a role in the development of atherosclerosis, but also involved in the pathogenesis of stroke [5]–[7]. Although inflammatory mechanisms in ischemic stroke have not been precisely clarified, several studies have reported that inflammation exerts major and complicated roles in the pathogenesis and prognosis of AIS. Meanwhile, various biomarkers of inflammation, such as homocysteine (Hcy) and C-reactive protein (CRP), have been explored association with AIS [5], [8]–[12]. Increased CRP was shown to be associated with poor prognosis when measured within 24 h of acute ischemic stroke [13], and elevated Hcy levels also have been found in stroke in previous studies [14], [15]. However, there has been little attention paid to the role of CRP and Hcy in the pathogenesis, progression and prognosis of BAD, which is common in Chinese AIS populations, but underused in clinical practice. Therefore, we conducted this study to investigate whether CRP and Hcy are associated with pathogenesis, progressive motor deficits and functional outcome in patients with BAD and (LD), respectively.

## Methods

### Study Population

We retrospectively recruited 1458 consecutive AIS patients admitted to Department of Neurology, the Third Affiliated Hospital of Sun Yat-sen University, Guangzhou, China, between January 2008 and January 2012. According to the radiological findings and previous report [16], intracranial BAD were diagnosed as follows: BAD of the paramedian pontine arteries (PPA) was defined as isolated unilateral infarcts extending to the ventral surface of the pons, while BAD in the lenticulostriate arteries (LSA) was defined as intracerebral infarcts with a diameter greater than 15 mm and visible as three or more lesions on an axial slice. Other infarcts that were not classified as BAD in either territory, of the lenticulostriate arteries or the anterior pontine arteries, were classified as LD. In our study, of the 1458 patients with AIS, 179 patients had BAD and 129 patients had LD. Both BAD and LD patients’ clinical data was analyzed.

### Clinical Data

Demographic and clinical data were collected from patients’ records, including age, gender, history of hypertension, dyslipidemia, diabetes mellitus, ischemic heart disease, smoking, National Institutes of Health Stroke Scale (NIHSS) scores on admission and within 5 days, and Modified Rankin Scale (mRS) at one month after symptoms onset.

Computed tomography (CT) scan was performed within 24 hours of admission and magnetic resonance imaging (MRI) was run within 72 hours of admission (General Electric, Milwaukee, WI, USA). All images were evaluated by two experienced neuroradiologists blinded to clinical data. All patients underwent a detailed history and routine examinations such as laboratory tests, electrocardiogram (ECG), chest X-rays, Doppler and color-coded Duplex sonography. Extracranial carotid artery and vertebral artery stenosis were evaluated by color-coded Duplex sonography, while the intracranial artery was assessed by magnetic resonance angiography (MRA).

Venous blood samples for CRP and Hcy measurement were obtained within 24 h of admission. The serum was separated by centrifugation at 3,000 rpm for 10 minutes and immediately stored at –70°C till being detected. The CRP level was measured by latex immunoturbidimetric assay according to Ichihara’s study [17], and the plasma level of Hcy was determined using a solid phase competitive chemiluminescent enzyme immunoassay [18].

### Exclusion Criteria

The exclude criteria included: (1) a potential source of cardiac embolism; (2) a significant (≥50%) stenosis or presence of vulnerable plaques of intracranial large arteries, extracranial carotid arteries or vertebral arteries; (3) absence of motor deficits; (4) infarcts were not located in the LSA or PPA distribution; (5) cortical infarcts, border-zone infarcts, or acute multiple infarcts shown by diffusion weighted imaging (DWI); (6) a history of vasculitis or dissection of the internal carotid artery; (7) a history of hemorrhagic stroke, coagulopathy, hematological disorders, receiving thrombolytic therapy or other endovascular interventions; (8) incomplete records; (9) interference of CRP level by any disorder, including asthma, arthritis, cancer, liver disease and a history of fever, cold, flu, urinary infection, bronchitis, sinus infection, pneumonia, tooth infection, gout flare or arthritis flare within the past two weeks.

### Unified Criteria

Hypertension was defined as either use of antihypertensive agents or systolic blood pressure (SBP) ≥140 mmHg and/or diastolic blood pressure (DBP) ≥ 90 mmHg, persisting at least three different occasions on three successive days after acute phase of ischemic stroke. Diabetes mellitus was defined as fasting plasma glucose ≥7.0 mmol/l, or a 2-hour plasma glucose≥11.1 mmol/l during an oral glucose tolerance test, or a random blood glucose ≥11.1 mmol/l combined with classical symptoms of hyperglycemia, a hypoglycemic crisis, or hemoglobin A1c >6.5%, or by treatment with oral hypoglycaemic drugs or insulin [19]. Dyslipidaemia was diagnosed as total serum cholesterol level (TC) ≥6.2 mmol/l or low-density lipoprotein cholesterol (LDL-C) ≥2.6 mmol/l or triglyceride (TG) ≥1.8 mmol/l, or was receiving cholesterol lowering drug. Smoking was defined as previous or current regular daily consumption of more than five cigarettes.

### Evaluation of progression

NIHSS was performed daily to trace the disease course within the first 5 days. Progression was defined as worsening by > or  = 1-point in the NIHSS for motor function during observation.

### Evaluation of prognosis

Functional outcome was evaluated by the modified Rank Scale (mRS) at 1 month after ictus. Good outcome was defined as mRS ≤ 2 and poor outcome was deemed as mRS > 2.

### Ethics Statement

This study was approved by the local Ethics Committee of the Third Affiliated Hospital of Sun Yat-sen University and conducted under the provisions of the Declaration of Helsinki. Informed consent for this investigation was obtained from all the patients or their family members for all experimental procedures that benefited the diagnosis and therapy. Consent was obtained from the family member in some cases when the patients had offered written authorization. The consent was verbal for measurements on NIHSS, mRS, homocysteine and C-reactive protein. This consent procedure was documented and the ethics committee was aware that this particular consent procedure would be obtained verbally. All these measurements were anonymously in this study. All the data were collected from an electronic system which was open to us when the agreements were got from the patients and ethics committee. Since these measurements benefit to the therapies of BAD and LD patients involved in this study, the ethics committee specifically approved that procedure and wish these measurements become a routine way for the treatment of BAD and LD patients in the future.

### Statistical Analysis

Numerical variables are reported as mean±standard deviation or median (interquartile range), and categorical variables are expressed as a percentage. Student t-test was used for normally distributed variables, Mann–Whitney U test for non-normally distributed variables were used to assess the differences between continuous variables, and the χ^2^ test or Fisher exact test for categorical variables. Logistic regression analysis was used to determine independent risk predictors. Meanwhile, the odds ratio (OR) and 95% confidence interval (CI) were obtained from the analysis. P <0.05 was considered statistically significant. SPSS version 16.0 software (Chicago, IL, USA) was used to perform for the aforementioned statistical analyses.

## Results

### Subject characteristics

**Figure 1:**
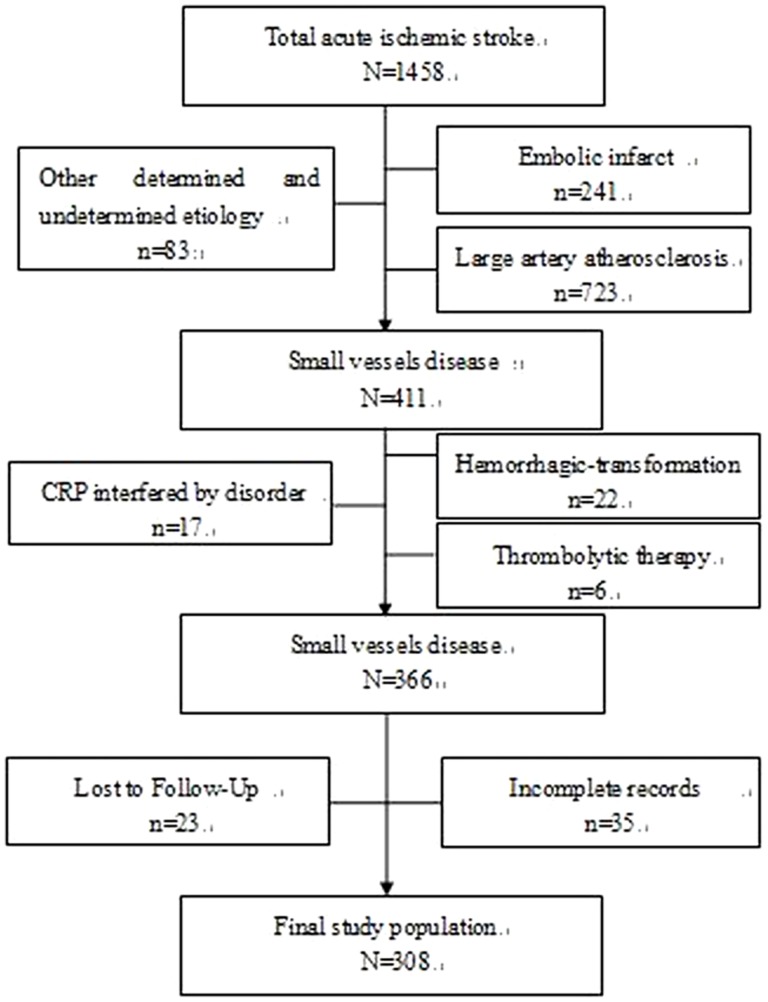
Inclusion and exclusion of subjects.

Of the 1458 inpatients diagnosed with AIS between January 2008 and January 2012, a cohort of 179 patients with BAD and 129 patients with LD enrolling in this retrospective study, met the inclusion criteria (Fig.1.).

The baseline characteristics of the patients with BAD and with LD are shown in Table 1. Compared with BAD, an obvious elevated Hcy level was observed in patients with LD (p = 0.030). Subjects with BAD had a significantly higher NIHSS score on admission (p<0.001) and mRS score at one month after ictus (p<0.001) than those with LD. Meanwhile, age, gender, CRP level, presence of hypertension, dyslipidemia, diabetes mellitus, smoking and ischemic heart disease did not differ between groups.

**Table 1:** Baseline characteristics of the 179 BAD patients and 129 LD patients.

	BAD	LD	P
	N = 179	N = 129	
Age, mean±SD	63.30±12.17	65.84±12.28	0.074
Gender, male, n (%)	121(67.6)	86(66.7)	0.864
CRP, median (IQR), mg/L	2.40(1.10–6.70)	2.71(1.30–5.60)	0.861
Hcy, mean±SD, umol/L	14.30±5.94	16.09±8.47	0.030*
Hypertension, n (%)	156(87.2)	116(89.9)	0.455
Dyslipidemia, n (%)	154(86.0)	103(79.8)	0.149
Diabetes mellitus, n (%)	77(43.0)	49(38.0)	0.376
Smoking, n (%)	56(31.3)	41(31.8)	0.926
Ischemic heart disease, n (%)	59(33.0)	56(43.4)	0.061
NIHSS on admission, median(IQR)	5 (3–7)	3 (1–5)	P<0.001**
mRS	2(1–3)	1(0–3)	P<0.001**

BAD, branch atheromatous disease; LD, lipohyalinotic degeneration; CRP, C-reactive protein; Hcy, Homocysteine; NIHSS, National Institutes of Health Stroke Scale; IQR, interquartile range; mRS, Modified Rankin Scale at one month after onset.

* P<0.05.

** P<0.01.

### Association between CRP/Hcy and progression of BAD and LD

Risk factors associated with progression in patients with BAD and LD were evaluated. Elevated Hcy (P = 0.004) and increased CRP (P = 0.025) were associated with progression in patients with BAD, but no difference of parameters was detected between progression and non-progression in patients with LD (Table 2).

**Table 2:** Comparison of progression and non-progression in patients with BAD and LD.

	BAD			LD		
	Progression	Non-progression	P	Progression	Non-progression	P
	N = 75	N = 104		N = 14	N = 115	
Age, mean±SD	64.31±13.32	62.58±11.29	0.363	68.50±12.71	65.43±12.21	0.379
Gender, male, n (%)	46(61.3)	75(72.1)	0.128	10(71.4)	76(66.1)	0.920
CRP, median (IQR), mg/L	2.9(1.7–8.4)	2.11(0.90–5.28)	0.025*	2.95(1.68–7)	2.7(1.26–5.2)	0.552
Hcy, mean±SD, umol/L	15.90±7.00	13.15±4.74	0.004**	15.75±6.17	16.14±8.73	0.872
Hypertension, n (%)	65(86.7)	91(87.5)	0.869	12(85.7)	104(90.4)	0.933
Dyslipidemia, n (%)	63(84.0)	91(87.5)	0.505	9(64.3)	94(81.7)	0.156
Diabetes mellitus, n (%)	37(49.3)	40(38.4)	0.147	6(42.9)	43(37.4)	0.691
Smoking, n (%)	23(30.7)	33(31.7)	0.880	3(21.4)	38(33.0)	0.564
Ischemic heart disease, n (%)	27(36.0)	32(30.8)	0.463	8(57.1)	48(41.7)	0.272

BAD, branch atheromatous disease; LD, lipohyalinotic degeneration; CRP, C-reactive protein; Hcy, Homocysteine; IQR, interquartile range; Progression, defined as worsening by ≥ 1 point in the NIHSS for motor function during the first 5 days.

* P<0.05.

** P<0.01.

Logistic regression analysis showed that significant association with progression of BAD was identified for elevated Hcy (OR: 1.103, 95% CI: 1.036–1.175), whereas CRP (OR: 1.019, 95% CI: 0.994–1.044) failed to show a statistically significant association after adjustment.

### Association between CRP/Hcy and prognosis of BAD and LD

As shown in Table 3, factors related to prognosis in patients with BAD and LD were analyzed, respectively. The comparison between poor outcome and good outcome in patients with BAD showed significant differences with respect to CRP (p = 0.006), and diabetes mellitus (p = 0.011). Interestingly, in patients with LD, we also found no difference between poor outcome and good outcome. After adjustment for age, gender, Hcy, hypertension, dyslipidemia, diabetes mellitus and ischemic heart disease, smoking (OR: 2.768, 95% CI: 1.247–6.145) and increased CRP (OR: 1.031, 95% CI: 1.004–1.060) were strong associated with poor outcome of BAD, while diabetes mellitus lost statistic significant association.

**Table 3:** Comparison of good outcome and poor outcome in patients with BAD and LD.

	BAD			LD		
	Good outcome	Poor outcome	P	Good outcome	Poor outcome	P
	N = 103	N = 76		N = 93	N = 36	
Age, mean±SD	62.94±11.76	63.79±12.77	0.646	65.38±12.52	66.78±11.66	0.562
Gender, male, n (%)	72(69.9)	49(64.5)	0.443	65(70.0)	21(58.3)	0.212
CRP, median (IQR), mg/L	2.1(0.9–5.06)	3.15(1.7–5.03)	0.006**	2.6(1.2–4.65)	2.8(1.42–7.93)	0.355
Hcy, mean±SD, umol/L	14.64±6.65	13.84±4.79	0.347	16.55±9.57	14.89±4.43	0.183
Hypertension, n (%)	87(84.5)	69(90.8)	0.211	83(89.2)	33(91.7)	0.934
Dyslipidemia, n (%)	90(87.4)	64(84.2)	0.546	73(78.5)	30(83.3)	0.539
Diabetes mellitus, n (%)	36(34.9)	41(53.9)	0.011*	36(38.7)	13(36.1)	0.785
Smoking, n (%)	27(26.2)	29(38.2)	0.088	32(34.4)	9(25.0)	0.303
Ischemic heart disease, n (%)	28(27.2)	31(40.8)	0.056	39(42.0)	17(47.2)	0.587

BAD, branch atheromatous disease; LD, lipohyalinotic degeneration; CRP, C-reactive protein; Hcy, Homocysteine; IQR, interquartile range; Good outcome, Modified Rankin Scale score ≤ 2 point at one month after onset; Poor outcome, Modified Rankin Scale score > 2 point at one month after onset.

* P<0.05.

** P<0.01.

### Characteristics of Progression and prognosis in subgroups of patients with BAD

Based on the results of association between Hcy/CRP and progression/prognosis of BAD, we then divided BAD into two common groups: LSA and PPA. In the LSA group, patients with progression had significantly higher levels of Hcy (P = 0.027) and CRP (P = 0.001) compared to those with non-progression patients; moreover, the level of Hcy (P = 0.035) was significantly higher, but the difference of CRP did not reach significance in the PPA group of patients with progression compared to non-progression. Patients in both two topographically identified groups with good outcome had a significantly lower level of CRP (P<0.01) than poor outcome, and the incidence of diabetes mellitus (P = 0.033) was significantly more prevalent in the PPA group of patients with poor outcome (Table 4).

**Table 4:** Comparison of progression and non-progression, good outcome and poor outcome in patients with BAD in the lenticulostriate arteries and paramedian pontine arteries, respectively.

	BAD					
	Progression	Non-progression	P	Good outcome	Poor outcome	P
LSA	N = 51	N = 66		N = 64	N = 53	
Age, mean±SD	64.20±12.90	61.50±12.21	0.250	61.54±12.04	63.83±13.43	0.338
Gender, male, n (%)	32(62.7)	47(71.2)	0.332	44(68.8)	33(62.3)	0.462
CRP, median (IQR), mg/L	3.20(1.87–9.20)	2.04(0.84–5.02)	0.001**	2.0(0.90–5.00)	3.20(1.73–9.00)	0.005**
Hcy, mean±SD, umol/L	16.04±6.65	13.63±5.02	0.027*	14.69±6.11	14.00±5.30	0.523
Hypertension, n (%)	45(88.2)	57(86.4)	0.764	54(84.4)	46(86.8)	0.712
Dyslipidemia, n (%)	45(88.2)	61(92.4)	0.652	58(90.6)	46(86.8)	0.511
Diabetes mellitus, n (%)	20(39.2)	22(33.3)	0.511	18(28.1)	24(45.3)	0.054
Smoking, n (%)	17(33.3)	19(28.8)	0.597	17(26.6)	18(34.0)	0.384
Ischemic heart disease, n (%)	19(37.2)	20(30.3)	0.429	15(23.4)	18(34.0)	0.208
PPA	N = 24	N = 38		N = 39	N = 23	
Age, mean±SD	64.54±14.44	64.45±9.33	0.977	65.15±11.09	63.71±11.51	0.621
Gender, male, n (%)	14(58.3)	28(73.7)	0.208	28(71.8)	16(69.6)	0.852
CRP, median (IQR), mg/L	1.98(0.80–5.49)	2.66(1.05–7.71)	0.492	2.35(0.90–5.30)	3.56(1.60–9.20)	0.003**
Hcy, mean±SD, umol/L	15.61±7.85	12.31±4.14	0.035*	14.57±7.51	13.48±3.55	0.510
Hypertension, n (%)	20(83.3)	34(89.5)	0.700	33(84.6)	23(100.0)	0.076
Dyslipidemia, n (%)	18(75.0)	30(78.9)	0.717	32(82.1)	18(78.3)	0.748
Diabetes mellitus, n (%)	17(70.8)	18(47.4)	0.070	18(46.2)	17(74.0)	0.033*
Smoking, n (%)	6(25.0)	14(36.8)	0.331	10(25.6)	11(47.8)	0.075
Ischemic heart disease, n (%)	8(33.3)	12(31.6)	0.886	13(33.3)	13(56.5)	0.074

BAD, branch atheromatous disease; LSA, lenticulostriate arteries; PPA, paramedian pontine arteries; CRP, C-reactive protein; Hcy, Homocysteine; IQR, interquartile range; Progression, defined as worsening by ≥ 1 point in the NIHSS for motor function during the first 5 days; Good outcome, Modified Rankin Scale score ≤ 2 point at one month after onset; Poor outcome, Modified Rankin Scale score > 2 point at one month after onset.

* P<0.05.

** P<0.01.

## Discussion

In this study, we found that elevated Hcy was an independent risk factor of progression in patients with BAD, meanwhile, increased CRP as well as history of smoking had potentials to independently forecast poor prognosis in patients with BAD. Whereas, no association between CRP/Hcy and progression/prognosis was observed in patients with LD. To the best of our knowledge, this is the first study conducted to systematically assess the association between CRP/Hcy and progression/non-progression, good outcome/poor outcome in Chinese AIS patients with BAD and LD, respectively.

In the evaluation of clinical characteristics, we found higher Hcy level in patients with LD than that in patients with BAD (table 1). The exact mechanisms remain unclear. Previous studies reported that Hcy was associated with cerebral small vessel disease (cSVD), including white matter lesions (WML) and lacunar infarcts (LIs) on magnetic resonance imaging (MRI) [20], [21]. These studies are also standing for our finding, though it is beyond our expectation. Our result may reflect a different pathogenesis for patients with BAD and LD. This finding also implies that elevated Hcy may suggest a likelihood of LD rather than BAD, and CRP may equally influence BAD and LD in Chinese AIS patients.

The present study provides new insights into the importance of inflammation in the progression and prognosis of patients with BAD. In this study, a relationship between CRP/Hcy and progression was found. That was patients with progression in both LSA and PPA group had a higher level of Hcy than non-progression, but only patients with progression in the LSA group had increased CRP (table 2, 4). However, Hcy was observed to be the only independent risk factor of progression after adjusting potential confounders, while CRP became insignificant (table 5). These findings are important, because Hcy level can be regulated by folic acid, vitamin B6 and B12 and could therefore be a potential target for therapy [22]. Previous study indicated that CRP level was independently associated with early neurologic deterioration [23], but in our study, we found CRP only related to progression of the LSA patients. One explanation for this inconsistency is that the study population of the previous research is different from our study, the former of which represented all of AIS patients. Notably, higher Hcy level is an independent predictor of progression in patients with BAD. The precise reasons remain unclear. However, Hcy may actively contribute to the progression and/or instability of the atheromatous plaque. Some previous studies suggested Hcy related to the development of microvascular disease via direct neurotoxicity, impairment of endothelial function and vascular smooth muscle [24], [25]. Future studies are needed to illustrate the accurate mechanism of progression related to Hcy in patients with BAD.

**Table 5:** Logistic regression analysis on BAD patients in facets of progression and Poor outcome.

Model	Variables	OR	95%CI for OR	p
1	CRP	1.019	0.994–1.044	0.138
	Hcy	1.103	1.036–1.175	0.002**
2	CRP	1.031	1.004–1.060	0.027*
	Diabetes mellitus	1.841	0.962–3.522	0.065
	Smoking	2.768	1.247–6.145	0.012*

BAD, branch atheromatous disease; CRP, C-reactive protein; Hcy, Homocysteine; Progression, defined as worsening by ≥ 1 point in the NIHSS for motor function during the first 5 days; Poor outcome, Modified Rankin Scale score > 2 point at one month after onset; OR, odds ratio; Model 1 =  progression; Model 2 =  poor outcome.

* P<0.05.

** P<0.01.

We found an elevated CRP level and diabetes mellitus were significantly associated with poor outcome in patients with BAD and its subgroup of PPA, while only the elevated CRP level had a relevance to that in the LSA group (table 3, 4). A previous study demonstrated that infratentorial infarcts were more common in elderly diabetic patients, suggesting the vulnerability of vertebrobasilar circulation in diabetes [26]. However, after adjustment for various potential confounders, the independent predictors of poor outcome in patients with BAD were CRP and smoking, while diabetes mellitus became insignificant (table 5). This phenomenon can be demonstrated by interaction between diabetes mellitus and CRP, hypertension, dyslipidemia. There are several probable reasons demonstrating the role of CRP in the prognosis of BAD. CRP is not only an inflammatory biomarker but also performs complex modulatory functions. It participates in maintaining and enhancing inflammation in cerebral vessels and brain injury by activation of complement cascade, initiation of leukocyte chemotaxis and expression of adhesion molecules through a positive feedback mechanism [27]–[29]. In addition, it induces apoptosis through a caspase-dependent mechanism [30]. These can delay or even exacerbate the recovery course of patients with BAD. The present study also showed no correlation between Hcy levels and functional disability at one month after the onset of stroke in patients with BAD, while another study reported that elevated Hcy levels have no significant association with the prognosis of functional disability [31].

There are several limitations in this study. First, this is a retrospective study. From this standpoint, possible selection bias may happen. However, to minimize this error, we restrict inclusion and exclusion criteria to avoid potential confounding factors. Second, this is a single-center research involving a modest sample size of Chinese AIS patients with BAD and LD. Multicenter studies with large and diverse cohorts as well as ethnicities need to be done. Last, this study only adopted CRP and Hcy as laboratory biomarkers on inflammation. However, the measurements of CRP and Hcy are of great use in clinical practice.

In conclusion, in patients with BAD, elevated Hcy may independently predict progression, and, increased CRP as well as history of smoking had potentials to independently predict poor prognosis. In addition, elevated Hcy might be associated with the pathogenesis in patients with LD. Further investigations are needed to explore the mechanisms behind this association.
